# Meta-analysis on the safety and efficacy of Erdafitinib in treating FGFR1–4 mutated solid tumors

**DOI:** 10.3389/fonc.2025.1571434

**Published:** 2025-08-08

**Authors:** Jiyu Huang, Zihan Wang, Fei Zhao, Adilai Aisa, Shengkai Tian, Siyuan Chen, Lianyi Peng, Xiaolu Yang, Jianxin He, Yue Yang

**Affiliations:** ^1^ Medical College, Northwest University for Nationalities, Lanzhou, China; ^2^ China Medical University, Shenyang, Liaoning, China; ^3^ Gansu University of Chinese Medicine, Lanzhou, Gansu, China; ^4^ Key Laboratory of Dunhuang Medicine and Transformation, Gansu University of Chinese Medicine, Lanzhou, Gansu, China

**Keywords:** erdafitinib, urothelial carcinoma, FGFR1-4, tumors, meta-analysis

## Abstract

**Objective:**

This article aims to analyze the safety and efficacy of Erdafitinib in the treatment of patients with advanced solid tumors harboring FGFR1–4 mutations.

**Methods:**

Search for relevant articles in databases such as PubMed, Embase, The Cochrane Library, Web of Science, and CNKI, covering the period from their establishment to October 25, 2024. Summarize the adverse drug reaction (AE) data, overall survival (OS), median progression-free survival (PFS), objective response rate (ORR), and other relevant data for patients with advanced solid tumors treated with Erdafitinib for FGFR1–4 mutations. Conduct a meta-analysis on the corresponding summarized data using the software Stata 18.0.

**Results:**

Through our search, we identified a total of 10 articles involving 1019 patients. In urothelial carcinoma, the most prevalent adverse reactions are hyperphosphatemia (78.5%), diarrhea (56.5%), and stomatitis (51.1%). The most frequently reported adverse reactions in other solid tumors are hyperphosphatemia (66.5%), dry mouth (48.5%), and diarrhea (44.9%). Patients with urothelial carcinoma treated with Erdafitinib exhibit higher median progression-free survival (PFS) and objective response rate (ORR) compared to those treated with other solid tumor therapies.

**Conclusion:**

Current evidence indicates that Erdafitinib exhibits certain therapeutic efficacy in the treatment of advanced solid tumors harboring FGFR1–4 mutations, with the most pronounced therapeutic effect observed in urothelial carcinoma. The efficacy of Erdafitinib in treating other solid tumors requires further confirmation through larger-scale studies involving a broader range of FGFR1–4 mutant tumors.

## Introduce

The fibroblast growth factor receptor (FGFR) family comprises four transmembrane receptor tyrosine kinases, namely FGFR 1-4, which can be triggered by over 20 known fibroblast growth factor (FGF) ligands, initiating crucial signaling cascades for cell proliferation, survival, angiogenesis, and differentiation (1). Alterations in FGFR1-4, encompassing gene mutations, specifically single nucleotide variations (SNVs), copy number amplifications, as well as gene rearrangements or fusions, are observed in roughly 5%-10% of human cancers. Erdafitinib, a selective Fibroblast Growth Factor Receptor inhibitor, has the potential to target tumors stemming from diverse epidermal growth factor receptor mutations (2). Erdafitinib is the first selective oral pan-FGFR inhibitor approved by the FDA in 2019. Currently, it is primarily used for treating advanced tumors or metastatic urothelial carcinoma (3). Urothelial bladder cancer is a heterogeneous epithelial malignancy, with the most common manifestation being an exophytic tumor confined to the mucosa or lamina propria (4). According to literature reports, urothelial cancer is primarily caused by alterations in FGFR2/3. Long-term follow-up after Erdafitinib treatment has confirmed its efficacy and safety (5). In advanced solid tumors, including glioma, thymic cancer, gastrointestinal cancer, gynecological cancer, and rare cancers, epidermal growth factor receptor alterations, which act as tumor markers, are also present. Erdafitinib treatment proves to be both effective and safe (6–12). There is an association between FGFR3 alterations and the response of urothelial carcinoma to chemotherapy and immunotherapy (13–18).

A previous meta-analysis by Zheng et al. (19) included only a small number of studies and did not explore subgroup differences. To build on that work, we added more recent studies and applied updated methods to better assess the efficacy and side effects of erdafitinib in different tumor types. We hope these findings can offer more practical insights for clinical treatment decisions.

## Materials and methods

### Literature search

The databases searched include PubMed, Embase, The Cochrane Library, Web of Science, and China National Knowledge Infrastructure(CNKI). The search timeframe spans from the databases’ establishment to October 25, 2024. Search terms: “erdafitinib”, “JNJ-42756493”, “Balversa”, “Receptors, Fibroblast Growth Factor”, “Receptors, FGF”, “FGF Receptor”, “Receptor, FGF”, “Heparin Binding Growth Factor Receptor”, “FGF Receptor Complexes”, “FGF Receptor Complex”,”Tumor”, “Cancer”. We searched for all potential studies containing these search terms.

Inclusion Criteria:

1. Population: Adult patients with advanced or metastatic solid tumors harboring confirmed FGFR1–4 alterations by molecular testing.
2. Intervention: Single-agent treatment with erdafitinib, regardless of dosing schedule or regimen.
3. Study design: Clinical trials of any design, including randomized controlled trials, non-randomized controlled trials, cohort studies, case–control studies, and single-arm trials.
4. Outcomes: Reported safety (e.g., incidence and severity of adverse events) and/or efficacy endpoints (e.g., objective response rate, progression-free survival, overall survival) for erdafitinib.

Exclusion criteria:

1. Patients with intermediate to advanced solid tumors with FGFR1–4 variants in combination with other diseases
2. Combined Erdafitinib treatment and other interventions such as pharmacologic or non-pharmacologic therapy
3. Clinical trial literature and repetitive publications for which complete data were not available
4. meta-analyses, letters, reviews, medical record reports, and conference papers
5. Preclinical studies, such as animal experiments, cellular experiments, etc.
6. Full text is not available.

### Quality assessment and data extraction

#### Quality assessment

The screening of literature is independently carried out by two researchers, who are responsible for literature screening, data extraction, and literature quality evaluation. The results are then verified by a third party. In case of any disagreement, the researchers discuss and resolve it together or have it arbitrated by the third party. Eliminate obviously irrelevant literature by reviewing the titles and abstracts, and then conduct a more thorough examination of the preliminarily screened literature by reading the full texts.

MINORS was selected for quality evaluation in the single-arm trial, and the first 8 items of the evaluation indexes of the MINORS scale were used for evaluation out of 16 points, of which: the purpose of the study was clear (2 points) consistency of the included patients (2 points) expected data collection (2 points) the endpoint indexes reflecting the purpose of the study (2 points) the objectivity of the endpoint indexes’ evaluation (2 points) the adequacy of the follow-up time (2 points) the failure rate was less than 5 percent (2 points). Whether the sample size was estimated (2 points) is categorized as 12 points or more 8–11 points 8 points or less in the order of low quality, moderate quality and high quality literature.

The Newcastle-Ottawa Scale (NOS) (20) was used to evaluate the quality of the randomized controlled trials. The NOS consists of 3 main components: selection of study participants (4 points), comparability between groups (2 points) and results (3 points). The NOS score is divided into 3 levels, such as 0-3, 4-6, 6-9, etc., which are in the order of low quality, medium and high quality literature. The specific included studies can be found in **Table 1** (Characteristics of Included Studies).

**Table 1:** Characterist of included studies.

Author(The primary author)	Year	Clinical trial information	Research design	Research stage	Sample size	Median age(Age)	Treatment	Treatment plan	Median follow-up period	Histology
J W F Catto (11)	2024	NCT04172675	Open-label, multicenter	I	49	69(37-86)	Erdafitinib	6 mg/day, following a 28 days cycle. There is no need to increase the dosage, and the medication can be continued for a maximum of 2 years.	13.4 months	High-risk non-muscle-invasive bladder cancer
Joon Oh Park (9)	2024	NCT02699606	An open-label study, One-arm, Test lla	II	35	54 (25; 78)	Erdafitinib	Orally consume 8 mg/day, and under pharmacodynamic guidance, increase the dosage to 9 mg every 28 days. Subsequently, consider increasing the dosage to 10 mg, following a seven-day medication/seven-day drug withdrawal cycle.	The median duration of treatment was 3.8 (Range:0.5-35.6 months)	Advanced solid tumor
Shubham Pant (10)	2023	NCT04083976	multicenter;One-arm;Phase 2;RAGNAR research	II	217	57 (48–64)	Erdafitinib	Adults and adolescents aged 15–17 years commence with 8 mg of Erdafitinib and may increase to 9 mg based on serum phosphate concentration measured 14 days prior to the first cycle. Adolescents aged 12–14 years who commence with 5mg of Erdafitinib may have their dosage increased to 6mg or further to 8mg based on serum phosphate concentrations measured during the first 14 days of Cycle 1 and the first 7 days of Cycle 2.	17.9 months	Advanced solid tumor
Jun Gong (12)	2024	NCT06308822	open-label, One-arm, Phase I study	I	35	60	Erdafitinib	Take Erdafitinib orally once daily for a continuous period of 28 days. During the first 14 days of the 28-day cycle, the starting dose of Erdafilinib is 8mg, taken orally once daily. On the 15th day of the first cycle, if the serum phosphate level is less than 5.5 mg/dL and there is no evident toxicity, the dose of Erdafitinib can be increased to 9 mg, taken orally once daily. If the serum phosphate level is between 5.5 and 7 mg/dL and there is no significant toxicity, continue administering the medication at 8 mg per day. If the serum phosphate level exceeds 7 mg/dL or significant toxicity is present, adjust the dosage for each regimen accordingly.	4.2(3~5.2)months	solid tumor
Arlene O Siefker-Radtke (21)	2022	NCT02365597	open-label, 非对照, Phase 2 BLC2001 study	II	101	67 (61–73)	Erdafitinib	Orally take Erdafitinib 8 mg/day, once a day, for 28 consecutive days, and increase the dosage to 9 mg/day under pharmacodynamic guidance.	11 months	Urothelial carcinoma
Yohann Loriot (5)	2023	NCT03390504	random allocation, multicenter	III	135	66 (32–85)	Erdafitinib	Oral administration of Erdafitinib at 8mg/day, with each cycle lasting 21 days, and the dosage is increased to 9mg on the 14th day under pharmacodynamic guidance.	15.9 months	Advanced or metastatic urothelial cancer
A O Siefker-Radtke (22)	2023	NCT03473743	random, open-label Phase III THOR study	III	175	67(44-86)	Erdafitinib	Oral administration of Erdafitinib at a dose of 8 mg per day, with a dose increase to 9 mg on day 14 based on pharmacodynamic guidance.	33 months	Advanced or metastatic urothelial cancer
Josep Tabernero (7)	2015	NCT01703481	open-label, multicenter(Increasing multi-dose group)	I	65	59 (27-75)	Erdafitinib	The increasing doses of 0.5 mg, 2 mg, 4 mg, 6 mg, 9 mg, and 12 mg constitute a 21-day cycle;When following a 28-day cycle, adopt an intermittent plan of seven days of medication followed by seven days of drug withdrawal (with dosages of 10 milligrams and 12 milligrams).	8-16weeks	Advanced solid tumor
Tomohiro Nishina (8)	2017	NCT01962532	open-label, multicenter, One-arm, Dose escalation	I	19	62.1	Erdafitinib	The increasing doses of 2 mg, 4 mg and 6 mg constitute a 21-day cycle;When following a 28-day cycle, adopt an intermittent plan of seven days of medication followed by seven days of drug withdrawal (with dosages of 10 milligrams and 12 milligrams).	12weeks	Advanced or refractory solid tumors
Rastislav Bahleda (6)	2019	NCT01703481	multicenter, Escalating multi-dose cohort	I	187	60 (21-84)	Erdafitinib	The increasing doses of 0.5 mg, 2 mg, 4 mg, 6 mg, 9 mg, and 12 mg constitute a 21-day cycle;When following a 28-day cycle, adopt an intermittent plan of seven days of medication followed by seven days of drug withdrawal (with dosages of 10 milligrams and 12 milligrams).	24weeks	Advanced or refractory solid tumors

### Data extraction

The extracted literature includes: year, sample size, disease type, adverse reactions (AE), overall survival (OS), median progression-free survival (PFS), and objective response rate (ORR). Additionally, the age distribution of the study population is considered. For all the included clinical trial studies.

### Statistical analysis

All statistical analyses in this study were conducted using Stata 18.0 software. Adverse reactions and objective response rate (ORR) were treated as binary variables, while overall survival (OS) and median progression-free survival (PFS) were considered continuous variables. Additionally, a forest plot was generated. Heterogeneity testing is conducted, using the heterogeneity index (I2) as the quantitative indicator. When I2 ≤ 50%, a fixed-effects model is selected. When I2 > 50%, indicating significant heterogeneity, a random-effects model is adopted. An effect size of p < 0.05 signifies statistical significance.

### Literature screening results

**Figure 1:**
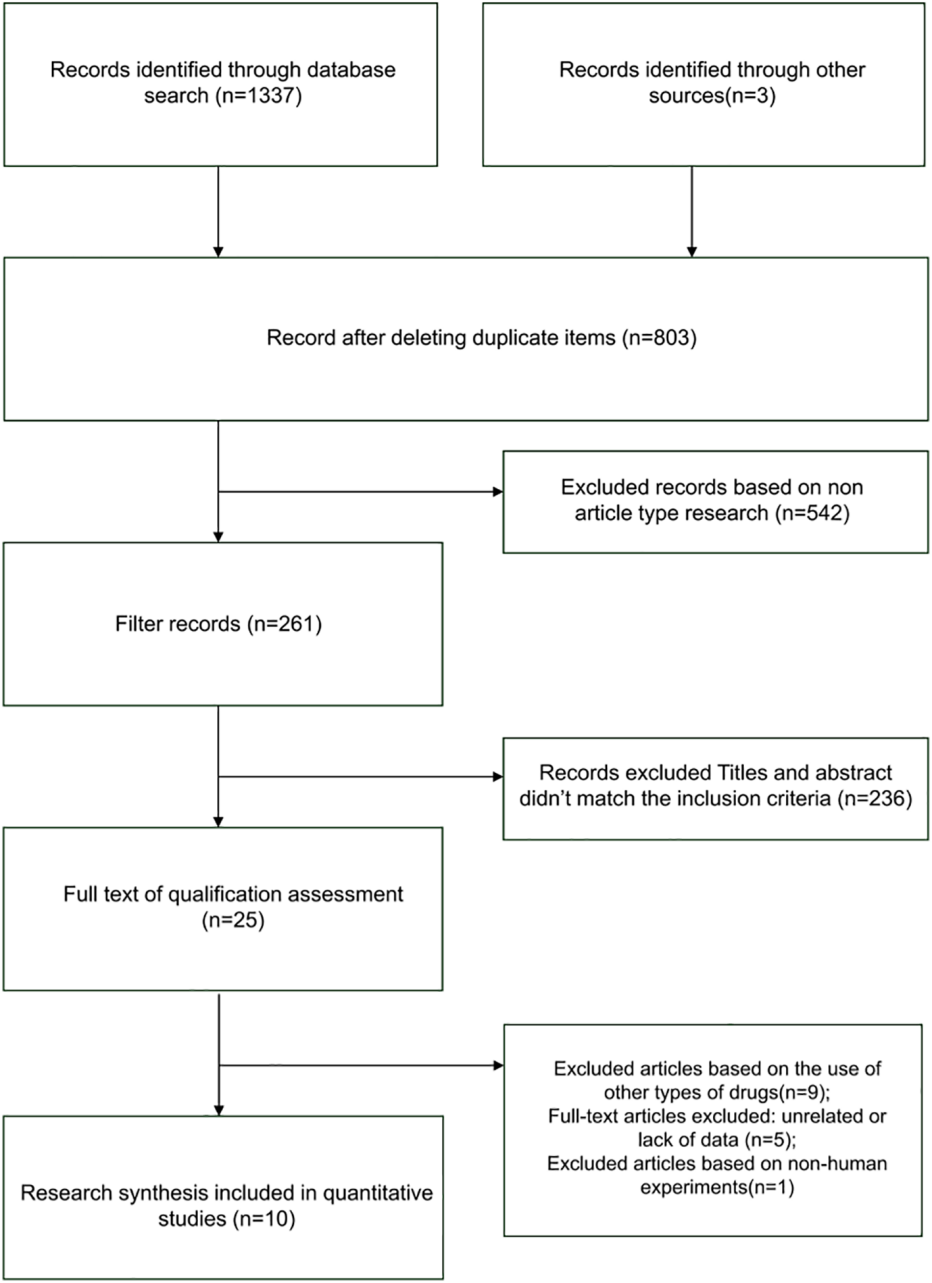
PRISMA flow diagram for study selection.

The literature screening process initially yielded 1337 relevant articles. After rigorous screening, 10 clinical trial studies involving 1019 patients were ultimately included. The literature screening process is illustrated in **Figure 1** below.

All the included studies, evaluated using the MINORS scale, had quality scores greater than 12 points. The NOS scale was evaluated and the quality scores of the included studies were all greater than 6.

### Calculate the 95% confidence interval for adverse drug reactions

Based on the included articles, this article categorizes the adverse drug reactions of Erafitinib in the treatment of FGFR1–4 mutant tumors into two subgroups: urothelial carcinoma and other solid tumors. The urothelial carcinoma group exclusively encompasses mid-to-late stage urothelial carcinoma, whereas the other solid tumors group comprises digestive system tumors, respiratory system tumors, reproductive system tumors, bone tumors, brain tumors, and more. The dosage of Erafitinib for both urothelial carcinoma and other solid tumors is approximately the same. The 95% confidence intervals for the overall adverse drug reactions for the articles included are shown in Annex S1.

Three articles were included in the urothelial carcinoma group, involving a total of 409 patients. Four articles were included in the other solid tumor group, involving a total of 334 patients.

## Result

The top three adverse reactions associated with urothelial carcinoma are hyperphosphatemia (78.5%), diarrhea (56.5%), and stomatitis (51.1%) (5, 21, 22). For other solid tumor groups, the corresponding top three adverse reactions are hyperphosphatemia (66.5%), dry mouth (48.5%), and diarrhea (44.9%) (9–12). In both groups, the incidence of adverse reactions such as eye diseases, skin and subcutaneous tissue diseases, neurological disorders, and enzyme imbalances exceeded 20%.

**Table 2** shows that hyperphosphatemia was the most common adverse reaction between the two groups. However, overall, the adverse reactions caused by Erafitinib in other solid tumor groups are slightly less severe than those observed in the urothelial carcinoma group.

**Table 2:** Summary of adverse events.

Urothelial carcinoma group						
Adverse event	Number of studies	Number of AE	Number of patients	AE event occurrence rate and 95% CI	P value	Model
Metabolic and nutritional disorders						
Hyperphosphatemia	3	321	409	0.785(0.744-0.824)	p = 0.000	Fixed model
Hyponatremia	1	24	173	0.139(0.091-0.199)	p = 0.000	Fixed model
Diseases of the blood and lymphatic systems						
Anemia	3	107	409	0.259(0.217-0.302)	p = 0.000	Stochastic Model
Eye diseases						
Dry eye syndrome	3	73	409	0.185(0.106-0.263)	p = 0.000	Stochastic Model
Blurred vision	1	18	101	0.178(0.109-0.267)	p = 0.000	Fixed model
Musculoskeletal and connective tissue diseases						
Joint pain	1	21	173	0.127(0.081-0.186)	p = 0.000	Fixed model
Back pain	1	20	173	0.116(0.072-0.173)	p = 0.000	Fixed model
Pain in the limbs	1	19	173	0.110(0.067-0.166)	p = 0.000	Fixed model
Gastrointestinal diseases						
Stomatitis	3	207	409	0.511(0.440-0.582)	p = 0.000	Stochastic Model
Diarrhea	3	231	409	0.565(0.516-0.613)	p = 0.000	Fixed model
Nauseated	3	70	409	0.170(0.135-0.209)	p = 0.000	Fixed model
Dry mouth	3	162	409	0.396(0.349-0.444)	p = 0.000	Fixed model
Decreased appetite	3	136	409	0.334(0.259-0.408)	p = 0.000	Stochastic Model
constipation	3	105	409	0.256(0.215-0.300)	p = 0.000	Fixed model
Vomiting	1	25	173	0.145(0.096-0.206)	p = 0.000	Fixed model
Abdominal pain	1	21	173	0.121(0.077-0.180)	p = 0.000	Fixed model
Diseases of the kidney and urinary system						
Urinary tract infection	2	41	274	0.149(0.109-0.194)	p = 0.000	Fixed model
hematuria	1	23	173	0.133(0.086-0.193)	p = 0.000	Fixed model
Diseases of the skin and subcutaneous tissue						
Hair loss	3	99	409	0.250(0.163-0.337)	p = 0.000	Stochastic Model
Dry skin	3	108	409	0.263(0.221-0.307)	p = 0.000	Fixed model
Nail detachment	1	28	135	0.207(0.142-0.307)	p = 0.000	Fixed model
Nail discoloration	2	53	308	0.172(0.132-0.217)	p = 0.000	Fixed model
Nail dystrophy	2	37	274	0.134(0.096-0.177)	p = 0.000	Fixed model
Palmoplantar Erythroderma Syndrome	3	104	409	0.253(0.212-0.297)	p = 0.000	Stochastic Model
Infections and general diseases						
Mumps	1	19	101	0.188(0.117-0.278)	p = 0.000	Fixed model
Weight loss	3	76	409	0.185(0.149-0.224)	p = 0.000	Fixed model
Fatigue	3	82	409	0.196(0.159-0.237)	p = 0.000	Fixed model
Inability	3	91	409	0.216(0.136-0.297)	p = 0.000	Stochastic Model
Diseases of the nervous system						
Limb paralysis	2	78	236	0.329(0.270-0.391)	p = 0.000	Fixed model
Taste disorders	1	42	173	0.243(0.181-0.314)	p = 0.000	Fixed model
Enzyme imbalance in the body (Investigations)						
Elevated alanine aminotransferase	3	84	409	0.205(0.142-0.278)	p = 0.000	Stochastic Model
Glycolysis	2	50	236	0.211(0.161-0.266)	p = 0.000	Fixed model
Elevated aspartate aminotransferase (AST)	2	57	308	0.184(0.143-0.230)	p = 0.000	Fixed model
Elevated serum creatinine	1	27	173	0.156(0.105-0.219)	p = 0.000	Fixed model
Other solid tumor groups						
Adverse event	Number of studies	Number of AE	Number of patients	AE event occurrence rate and 95% CI	P value	Model
Metabolic and nutritional disorders						
Hyperphosphatemia	4	230	334	0.665(0.475-0.832)	p = 0.000	Stochastic Model
Hyponatremia	1	25	217	0.115(0.076-0.165)	p = 0.000	Fixed model
Diseases of the blood and lymphatic systems						
Thrombocytopenia	2	25	250	0.096(0.061-0.138)	p = 0.000	Fixed model
Decreased lymphocyte count	1	5	33	0.152(0.051-0.319)	p = 0.000	Fixed model
Anemia	2	62	250	0.245(0.192-0.301)	p = 0.000	Fixed model
Eye diseases						
Dry eye syndrome	4	71	334	0.209(0.166-0.256)	p = 0.000	Fixed model
Blurred vision	1	27	217	0.124(0.084-0.176)	p = 0.000	Fixed model
Central chorioretinopathy	1	10	49	0.204(0.102-0.343)	p = 0.000	Fixed model
Musculoskeletal and connective tissue diseases						
Limb pain	1	25	217	0.115(0.076-0.165)	p = 0.000	Fixed model
Joint pain	2	45	252	0.176(0.130-0.226)	p = 0.000	Fixed model
Back pain	1	22	217	0.101(0.065-0.149)	p = 0.000	Fixed model
Myalgia	1	22	217	0.101(0.065-0.149)	p = 0.000	Fixed model
Gastrointestinal disorders						
Dry mouth	4	162	334	0.485(0.431-0.539)	p = 0.000	Fixed model
Stomatitis	4	166	334	0.442(0.320-0.568)	p = 0.000	Stochastic Model
Diarrhea	4	176	334	0.449(0.294-0.609)	p = 0.000	Stochastic Model
Constipation	4	92	334	0.241(0.144-0.354)	p = 0.000	Stochastic Model
Anorexia	4	87	334	0.258(0.211-0.307)	p = 0.000	Fixed model
Vomiting	2	46	250	0.182(0.135-0.230)	p = 0.000	Stochastic Model
Nauseated	2	49	250	0.193(0.145-0.246)	p = 0.000	Fixed model
Abdominal pain	1	34	217	0.157(0.111-0.212)	p = 0.000	Fixed model
Diseases of the skin and subcutaneous tissue.						
Dry skin	4	105	334	0.277(0.186-0.377)	p = 0.000	Stochastic Model
Nail dystrophy	2	38	266	0.315(0.258-0.375)	p = 0.000	Fixed model
Nail discoloration	2	42	252	0.160(0.115-0.205)	p = 0.000	Stochastic Model
Palmoplantar Erythroderma Syndrome	2	80	252	0.315(0.258-0.375)	p = 0.000	Fixed model
Nail detachment	2	24	84	0.213(0.130-0.309)	p = 0.000	Fixed model
Hair loss	2	53	266	0.198(0.151-0.249)	p = 0.000	Fixed model
Infections and general diseases						
Mumps	2	50	252	0.196(0.148-0.248)	p = 0.000	Fixed model
Fatigue	3	83	285	0.265(0.188-0.343)	p = 0.000	Fixed model
Feeble	1	28	217	0.129(0.087-0.181)	p = 0.000	Fixed model
Fever	1	25	217	0.115(0.076-0.165)	p = 0.000	Fixed model
Headache	1	22	217	0.101(0.065-0.149)	p = 0.000	Fixed model
Urinary tract infection	1	9	49	0.184(0.088-0.320)	p = 0.000	Fixed model
Diseases of the nervous system						
Limb paralysis	4	61	334	0.179(0.139-0.224)	p = 0.000	Fixed model
Taste disorders	1	11	49	0.224(0.118-0.366)	p = 0.000	Fixed model
Enzyme imbalance in the body(Investigations)						
Elevated alanine aminotransferase	3	83	285	0.290(0.164-0.435)	p = 0.000	Stochastic Model
Elevated aspartate aminotransferase (AST)	3	80	285	0.291(0.195-0.397)	p = 0.000	Stochastic Model
Elevated blood alkaline phosphatase	2	40	250	0.157(0.113-0.206)	p = 0.000	Fixed model

### Objective response rate of urothelial carcinoma (objective response rate)

**Figure 2:**
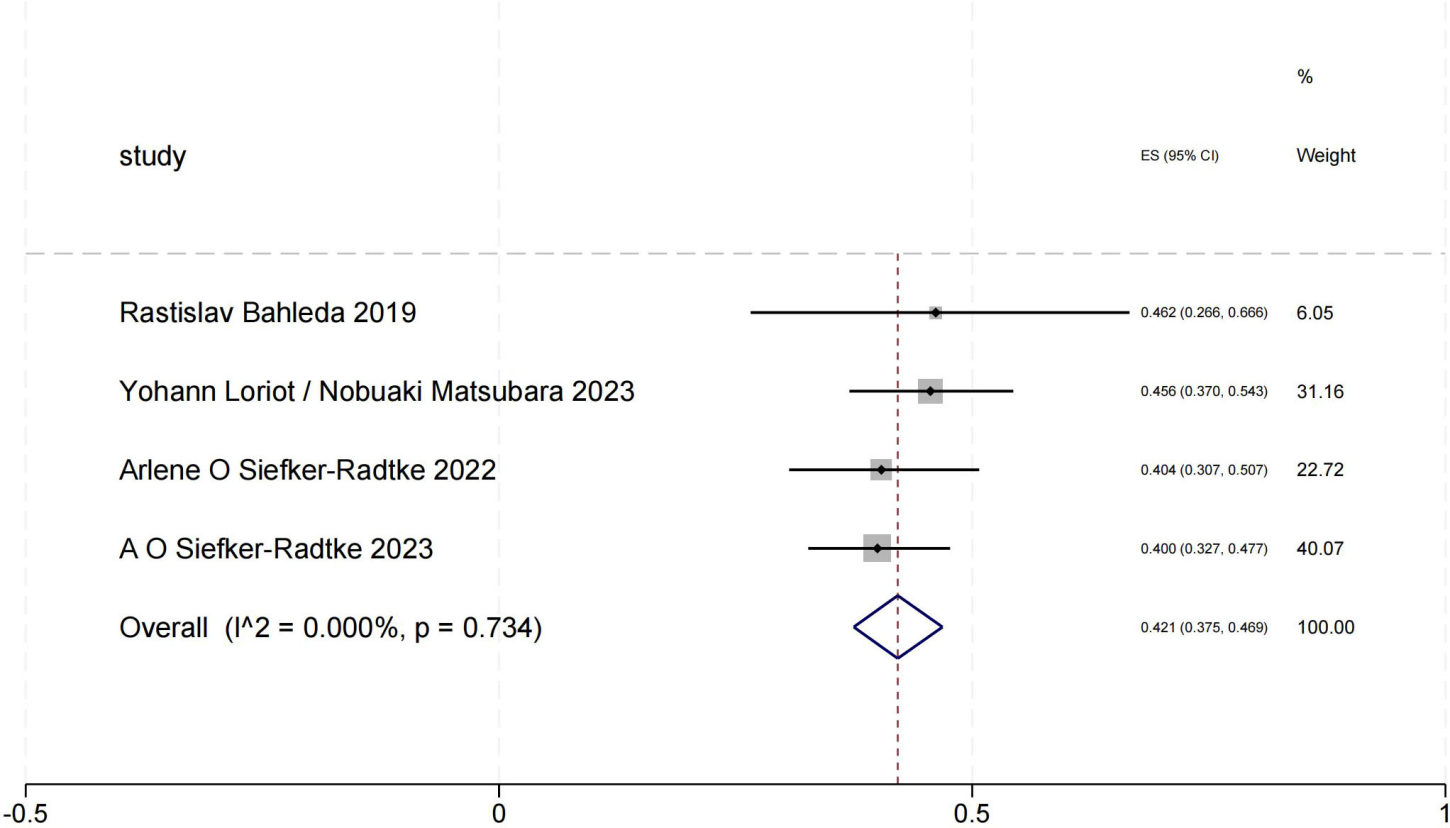
Objective response rate of urothelial carcinoma.

The ORR forest plot for urothelial carcinoma illustrates the 95% confidence intervals of ORR from four studies, providing a comprehensive analysis of each study’s results and assessing overall effects and heterogeneity. We obtained a total ORR value of 0.421 (95% CI:0.375-0.469). In this **Figure 2**, the heterogeneity among the four studies is minimal, indicating high consistency of the results.

### Overall survival of urothelial carcinoma(overall survival)

**Figure 3:**
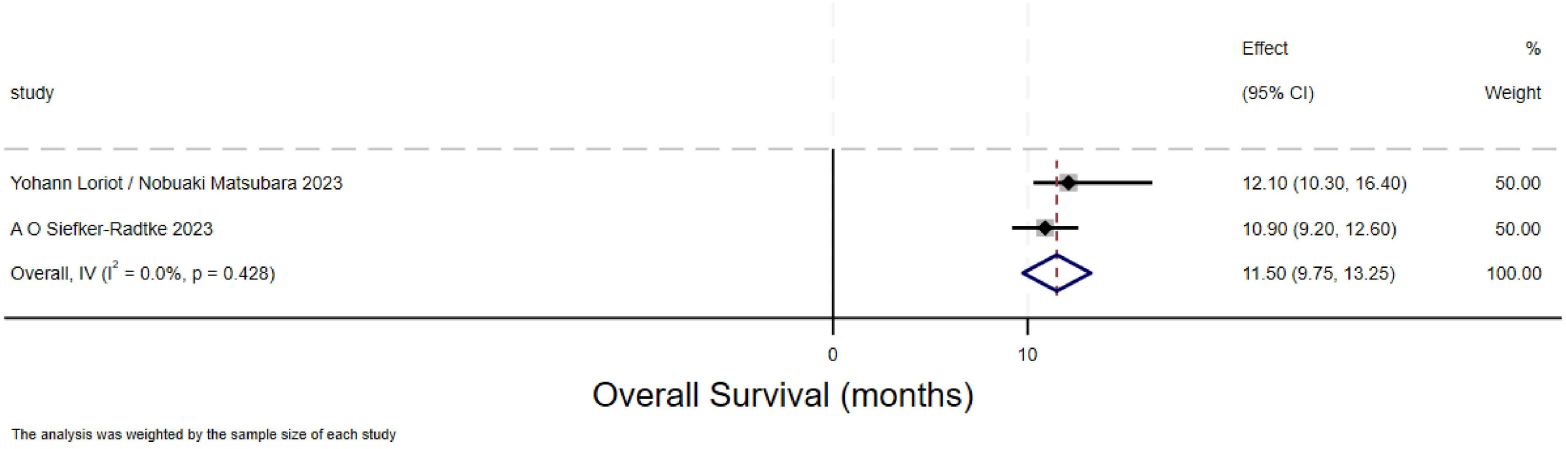
Overall survival of urothelial carcinoma.

The forest plot for overall survival (OS) in urothelial carcinoma illustrates the 95% confidence intervals for OS from two studies, providing a comprehensive analysis of the individual study results and assessing the overall effect and heterogeneity. The overall survival (OS) rate for urothelial carcinoma was 11.5 months (95% CI:9.75-13.25).Two methods are used to calculate the overall effect size respectively. The heterogeneity between the two studies depicted in this **Figure 3** is significant.

### Progression-free survival time for urothelial carcinoma(progression-free-survival)

**Figure 4:**
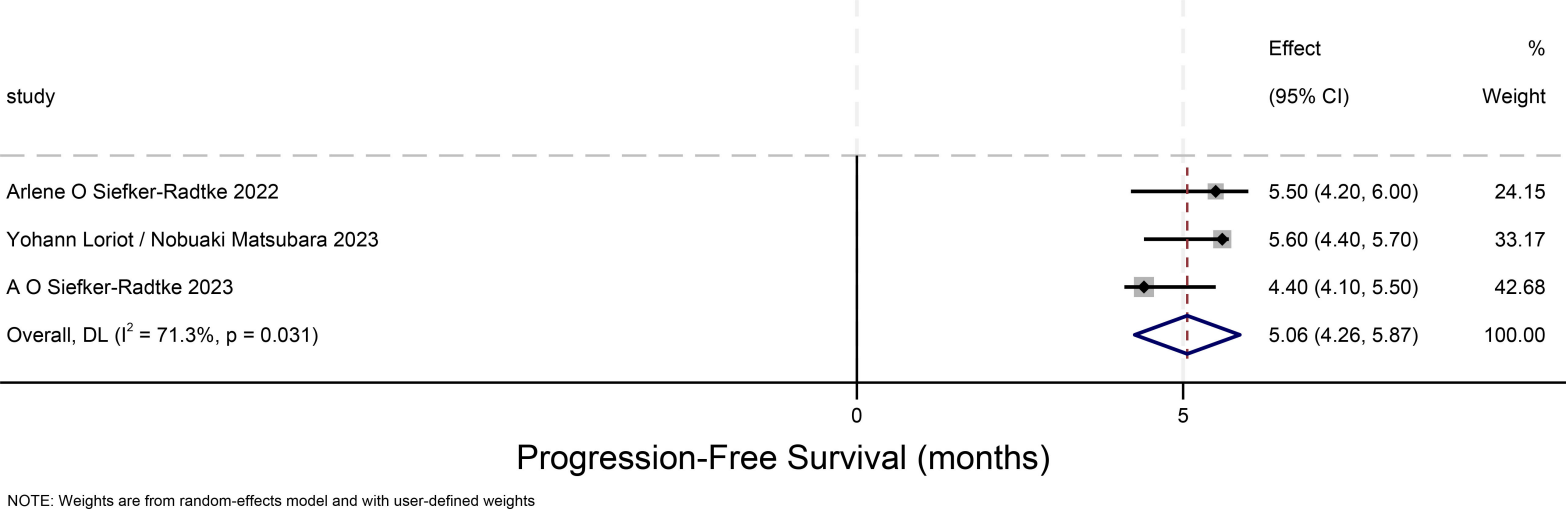
Progression-free survival time for urothelial carcinoma.

The PFS forest plot for urothelial carcinoma illustrates the 95% confidence intervals for PFS from three studies, providing a comprehensive analysis of the individual study results and assessing overall effects and heterogeneity. The weight proportion of each study is approximately 30%. The overall PFS for urothelial carcinoma is 5.06 months (95% CI:4.26-5.87), and there is considerable heterogeneity among the three studies depicted in this **Figure 4**.

### Objective response rate for other solid tumors(objective response rate)

**Figure 5:**
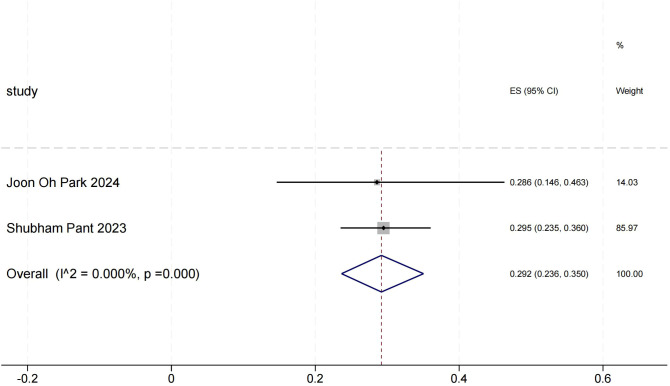
Objective response rate for other solid tumors.

The ORR forest plot for other solid tumors illustrates the 95% confidence intervals of ORR for two studies, providing a comprehensive analysis of each study’s results and assessing overall effects and heterogeneity. Shubham Pant’s research carries a significant weight. In **Figure 5**, the heterogeneity between the two studies is minimal, indicating high consistency in their results.

### Progression-free survival in other solid tumors(progression-free-survival)

**Figure 6:**
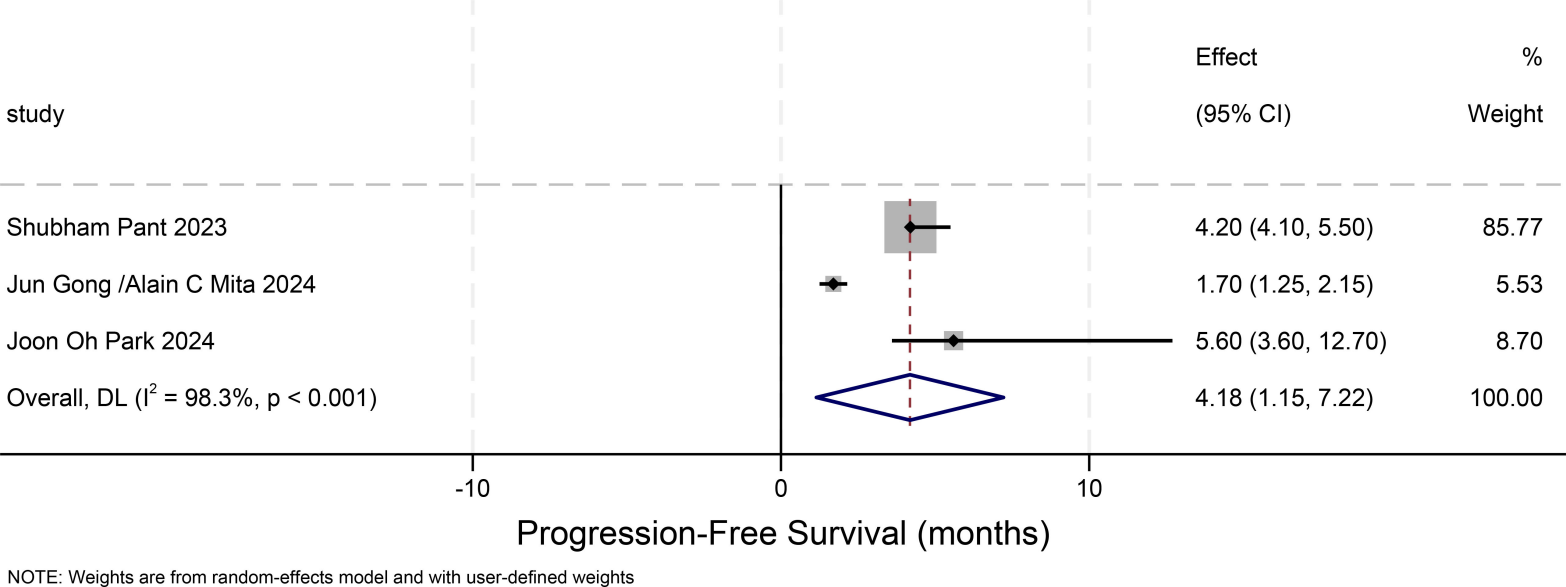
Progression-free survival in other solid tumors.

**Figure 6** illustrates the 95% confidence intervals of PFS from three studies on other solid tumors. A comprehensive analysis of the results from these three studies was conducted to evaluate the overall effect and heterogeneity. The 95% confidence intervals were not directly reported in the original study. Therefore, we approximated the 95% CIs by converting the available 90% CIs under the assumption of symmetry. The standard error was estimated as the half-width of the 90% CI divided by 1.645, and the 95% CI was then calculated as SE × 1.96. Final limits were rounded to the nearest 0.05. These values should be interpreted as approximate estimates. The PFS for other solid tumors is 4.18 months (95% CI: 1.15-7.22). Based on the findings of this study, it is evident that the majority of the selected patients had undergone three or more distinct types of treatments. Consequently, various treatment approaches might potentially disrupt the efficacy and adverse effects of erdafitinib, leading to inconsistencies compared to other research. As depicted in this figure, the heterogeneity among the three studies is excessively high, resulting in poor consistency of the outcomes.

## Discussion

Through literature research, this article explores the mechanism behind adverse reactions caused by erdafitinib and outlines preventive measures, aiming to enhance patient medication safety in the future.

Under physiological conditions, FGF23 initiates the mitogen-activated protein kinase (MAPK) signaling cascade through binding to fibroblast growth factor receptors (FGFRs) and Klotho (KL) coreceptor complexes. This signaling pathway enhances renal phosphate excretion while simultaneously upregulating Cyp24a1 expression and downregulating Cyp27b1 expression, ultimately reducing gastrointestinal phosphate absorption via decreased 1,25-dihydroxyvitamin D (1,25D) levels. Erdafitinib disrupts these dual regulatory mechanisms by inhibiting both pathways, thereby impairing physiological phosphate homeostasis through two distinct effects: 1) compromised renal phosphate elimination and 2) sustained intestinal phosphate absorption. This dual pharmacological interference culminates in the development of hyperphosphatemia (23). Although Erdafitinib can induce hyperphosphatemia, this condition is reversible. The adoption of interval medication, as described in Josep Taberneroe’s article, can significantly reduce the occurrence of hyperphosphatemia (7). In the CBGJ398X2204 study involving another FGFR inhibitor, Inflatinib, no patients discontinued treatment due to hyperphosphatemia. The majority of patients (81%) either took prophylactic phosphate binders (48%) or consumed phosphate binders after their first dose of Inflatinib (32%), significantly reducing the incidence of hyperphosphatemia (24).

Gastrointestinal adverse reactions are common side effects associated with the use of erdafitinib. Diarrhea may be mediated by epidermal growth factor receptor 4 signaling, and epidermal growth factor receptor inhibitors exhibit varying degrees of selectivity towards this signaling pathway. Inhibiting FGF19/FGFR4-mediated signaling upregulates the conversion of cholesterol to bile acids in the liver, leading to alterations in bile acid metabolism (25). It has been proven that an imbalance in bile acid metabolism can increase intestinal water secretion, enhance mucosal permeability, stimulate intestinal peristalsis, and ultimately lead to diarrhea (26).

Central serous chorioretinopathy is also one of the adverse reactions associated with the use of erdafitinib. Mild to moderate cases of central serous chorioretinopathy induced by erdafitinib may resolve with dose interruption or reduction, but occasionally, drug discontinuation is still necessary for relief. Alkaline FGFR is a neurotrophic factor, with the highest expression levels in the nuclei of macroglial cells and the retinal pigment epithelium (RPE). Erdafitinib, on the other hand, is an inhibitor of FGFR (27). Within cells, FGFR activates two intracellular transduction pathways: the mitogen-activated protein kinase (MAPK pathway, RAS/RAF/MEK/ERK pathway) and the phosphoinositide 3-kinase (PI3K pathway, PI3K/AKT/mTOR pathway), playing a pivotal role in preventing cell apoptosis (28–30). The MAPK pathway also regulates the tight junctions between RPE cells and exerts a regulatory effect on the fluid transport channel aquaporin 1 (AQP1). Consequently, inhibiting MAPK could potentially disrupt normal fluid transport, resulting in subretinal fluid accumulation (31) Therefore, the accumulation of subretinal fluid largely depends on the dosage of FGFR inhibitors used. In fact, upon interruption of erdafitinib treatment, fluid accumulation rapidly returns to normal, indicating a dose-dependent relationship between the dosage of erdafitinib and its ocular toxicity.

The use of FGFR inhibitors and other tyrosine kinase inhibitors can lead to skin lesions, which are distinct from the hand-foot syndrome induced by cytotoxic drugs like capecitabine and doxorubicin. Skin conditions resulting from the use of FGFR inhibitors manifest as focal calluses, hyperkeratosis, erythema, and fissures, primarily affecting the fingers and toes (32). In a systematic review encompassing 58 targeted drugs, it was reported that the use of FGFR inhibitors led to dry skin in 18% of patients. Common manifestations included itching, fine flakes, and cracks, which may progress to dry dermatitis. Additionally, there was an incidence of co-infection with Staphylococcus aureus, herpes simplex, or other bacterial and viral infections (33) Although this complication rarely leads to critical illness or death, the occurrence of mild xerosis may affect the overall efficacy of drug therapy (34).

It’s worth noting that in a study focusing on the treatment of advanced or metastatic urothelial carcinoma, it was discovered that 4% of patients developed hypercalcemia as an adverse reaction, whereas no such adverse reactions were observed in other research. Hypercalcemia, frequently induced by malignant tumors, is a prevalent symptom among advanced cancer patients, occurring in approximately 20% to 30% of cases, particularly among those with solid tumors and hematological malignancies (35) Hypercalcemia is also associated with a poor prognosis in cancer patients. The underlying mechanisms involve abnormalities in bone resorption, intestinal absorption, or renal excretion, resulting in abnormal calcium utilization. This can lead to symptoms across multiple systems, including gastrointestinal and neurological symptoms (36–38). The soluble protein RANKL, a member of the TNF family, is synthesized by osteoblasts and T cells, and plays a pivotal role in guiding the differentiation and activation of osteoclasts. It serves as a crucial regulatory factor for osteoclast formation, activity, and survival (39). Hypercalcemia, triggered by abnormal bone resorption, can be exacerbated by an increased release of RANKL, thereby enhancing osteoclast activity in tumor cells within the bone. This phenomenon is closely linked to the presence of bone metastasis in patients suffering from advanced urothelial carcinoma.

By administering erdafitinib in intervals and at low doses, we can achieve the objective of reducing its adverse reactions (9). Alternatively, combining erdafitinib with other medications, such as fluconazole (a moderate CYP2C9 and CYP3A inhibitor) and itraconazole (a potent CYP3A4 and P-gp inhibitor), can enhance the metabolic rate of Erdafitinib in the body, thus reducing the likelihood of adverse reactions (40).

Although erdafitinib has demonstrated significant efficacy in urothelial carcinoma with FGFGR mutations, certain patients still exhibit drug resistance or suboptimal therapeutic responses. To expand multimodal treatment approaches, emerging studies have revealed that neoadjuvant RC48-ADC combined with immune checkpoint inhibitors (ICIs) shows remarkable clinical benefits in locally advanced or metastatic muscle-invasive bladder cancer (MIBC) patients. Analysis of post-treatment gene expression profiles revealed significant overexpression of HSPA1A across all molecular subgroups, particularly in the C2 subtype, suggesting its critical association with therapeutic resistance (41, 42). Concurrently, HER2 - the primary target of RC48-ADC - demonstrated marked heterogeneity in spatial distribution. These findings collectively indicate that RC48-ADC represents a promising therapeutic strategy, particularly for patients resistant to immunotherapy or conventional chemotherapy, thereby offering potential for improved clinical outcomes. This evidence substantiates further evaluation of neoadjuvant RC48-ADC combined with ICIs in MIBC patients to optimize treatment algorithms (43).

Additionally, novel predictive models based on pretreatment clinical characteristics have been developed for MIBC treatment response assessment. Recent investigations have further elucidated the therapeutic advantages of tislelizumab-containing neoadjuvant combination regimens in MIBC management (44). These advancements collectively provide alternative therapeutic paradigms for MIBC patients, addressing the critical need for personalized treatment strategies in urothelial carcinoma management.

Shortcoming: Due to the limitation of retrieved articles, only 3 RCTs and 7 single-arm clinical studies were included, which lacked a large sample of high-quality RCTs. During the study, we only distinguished uroepithelial carcinoma from other solid tumors, and were unable to perform a multisubgroup analysis.

Tumor FGFR1–4 gene mutations are seldom isolated to a single mutation, and FGFRS gene mutations exhibit significant variations across different tumors, thus making clinical data collection challenging (45). Due to variations in tumor types, there exist disparities in the dosages of drugs administered. The dosage of Erdafitinib was not strictly controlled across experimental groups in various articles; however, we have endeavored to establish a reasonable dosage range (46).

The efficacy of Erdafitinib in treating urothelial carcinoma is notably superior to that in other FGFR-mutated tumors. This may be attributed to the differing impacts of FGFR mutations on various tumors (47). Meanwhile, the adverse reactions associated with Erdafitinib treatment for urothelial carcinoma are also more pronounced than those observed in other tumor types, although current research on this conclusion remains inconclusive (48)

Since erdafitinib was approved by the FDA in 2019 as the first oral pan-FGFR inhibitor and is currently only indicated for urothelial carcinoma, studies on other solid tumors remain limited, highlighting the need for more high-quality clinical data in non-urothelial cancers.

The scope of the literature is limited to English and Chinese, excluding other languages.

In summary, Erdafitinib is effective and safe in treating patients with FGFR1–4 mutations, providing evidence-based medicine for clinical medication. However, there are currently limited individual experimental studies focusing on tumors other than urothelial carcinoma. Longer and larger-scale trials are warranted to evaluate the efficacy and safety of Erdafitinib in the clinical application for treating other tumors.

## Data Availability

The original contributions presented in the study are included in the article/**Supplementary Material**. Further inquiries can be directed to the corresponding author.
